# Population Pharmacokinetic Properties of Sulfadoxine and Pyrimethamine: a Pooled Analysis To Inform Optimal Dosing in African Children with Uncomplicated Malaria

**DOI:** 10.1128/AAC.01370-17

**Published:** 2018-04-26

**Authors:** Miné de Kock, Joel Tarning, Lesley Workman, Elizabeth N. Allen, Mamadou M. Tekete, Abdoulaye A. Djimde, David J. Bell, Steve A. Ward, Karen I. Barnes, Paolo Denti

**Affiliations:** a Division of Clinical Pharmacology, Department of Medicine, University of Cape Town, Cape Town, South Africa; b Mahidol-Oxford Tropical Medicine Research Unit, Faculty of Tropical Medicine, Mahidol University, Bangkok, Thailand; c Centre for Tropical Medicine and Global Health, Nuffield Department of Medicine, University of Oxford, Oxford, United Kingdom; d Worldwide Antimalarial Resistance Network (WWARN), Oxford, United Kingdom; e Malaria Research and Training Center, Faculty of Pharmacy and Faculty of Medicine and Dentistry, University of Sciences, Technique and Technology of Bamako, Bamako, Mali; f Infectious Diseases Unit, Queen Elizabeth University Hospital, Glasgow, United Kingdom; g Molecular and Biochemical Parasitology Group, Liverpool School of Tropical Medicine, Liverpool, United Kingdom

**Keywords:** Monolix, malaria, modeling, pharmacokinetics, pharmacometrics

## Abstract

Sulfadoxine-pyrimethamine with amodiaquine is recommended by the World Health Organization as seasonal malaria chemoprevention for children aged 3 to 59 months in the sub-Sahel regions of Africa. Suboptimal dosing in children may lead to treatment failure and increased resistance. Pooled individual patient data from four previously published trials on the pharmacokinetics of sulfadoxine and pyrimethamine in 415 pediatric and 386 adult patients were analyzed using nonlinear mixed-effects modeling to evaluate the current dosing regimen and, if needed, to propose an optimized dosing regimen for children under 5 years of age. The population pharmacokinetics of sulfadoxine and pyrimethamine were both best described by a one-compartment disposition model with first-order absorption and elimination. Body weight, age, and nutritional status (measured as the weight-for-age Z-score) were found to be significant covariates. Allometric scaling with total body weight and the maturation of clearance in children by postgestational age improved the model fit. Underweight-for-age children were found to have 15.3% and 26.7% lower bioavailabilities of sulfadoxine and pyrimethamine, respectively, for each Z-score unit below −2. Under current dosing recommendations, simulation predicted that the median day 7 concentration was below the 25th percentile for a typical adult patient (50 kg) for sulfadoxine for patients in the weight bands of 8 to 9, 19 to 24, 46 to 49, and 74 to 79 kg and for pyrimethamine for patients in the weight bands of 8 to 9, 14 to 24, and 42 to 49 kg. An evidence-based dosing regimen was constructed that would achieve sulfadoxine and pyrimethamine exposures in young children and underweight-for-age young children that were similar to those currently seen in a typical adult.

## INTRODUCTION

While substantial progress has been made in recent years to lower mortality rates, malaria remained the fourth leading cause of death in sub-Saharan children under the age of 5 years in 2015, with a child dying from malaria every 2 min (1). Children are particularly vulnerable because, in areas of moderate- to high-intensity transmission, immunity to severe malaria is generally acquired by the age of 5 years and immunity to uncomplicated malaria is only attained in early adulthood (2).

Malaria treatment outcomes depend on several factors, including levels of parasite resistance to antimalarial drugs, host factors such as acquired immunity, and the pharmacokinetic (PK) properties of the antimalarial treatment. As age and acute malaria may alter the pharmacokinetic properties of most antimalarial drugs, studies in healthy adult volunteers are not sufficient for determining dosing regimens in children (3).

Sulfadoxine-pyrimethamine with amodiaquine is recommended by the WHO as seasonal malaria chemoprevention (SMC) in the Sahel subregion of Africa for areas with highly seasonal malaria transmission, where Plasmodium falciparum is sensitive to both antimalarial medicines. A full treatment course of sulfadoxine-pyrimethamine with amodiaquine is administered to children of 3 to 59 months of age at monthly intervals during the malaria season (1).

The disposition of sulfadoxine-pyrimethamine in children is poorly understood, even though the drugs have been used widely for over 50 years. Available data suggest the occurrence of suboptimal dosing in children (3). Dosing of antimalarials, such as sulfadoxine-pyrimethamine, has often been based on age for practical reasons, but this may lead to under- or overdosing (4).

PK studies are gaining recognition as tools to inform antimalarial drug policies and dosing regimens. Traditional PK data analysis requires multiple samples per patient, which can be challenging, particularly for small ill children. Population PK modeling requires less intensive sampling and can estimate PK parameters at the population and study arm levels while accounting for individual differences. This method does well in studying the nature of antimalarial drugs in children (5, 6).

There is uncertainty regarding the precise PK determinants of treatment outcome for malaria. There is evidence suggesting that the day 7 drug concentration (*C*_day7_) is a good determinant of outcome. The period between dosing and day 7 is crucial because it determines whether the parasite population is eliminated or causes recrudescence, assuming that drug concentrations have been above the day 7 level for 7 days (four 48-h parasite life cycles) (7). Toxicity is most likely related to the maximum concentration of the drug (*C*_max_), but no threshold for toxicity has been reported for sulfadoxine-pyrimethamine.

In this work, we present a pooled population pharmacokinetic analysis of data from four African studies (3, 5, 8, 9). The aims of this study were to (i) characterize the pharmacokinetic parameters of sulfadoxine-pyrimethamine by comparing young children to adults, using nonlinear mixed-effects modeling; (ii) explore the effects of predefined covariates, including nutritional status; and (iii) if needed, use simulation to optimize dosing in young children.

## RESULTS

### Data.

Pharmacokinetic data were collected for 801 patients, 415 of whom were children (Table 1). A total of 259 of 8,981 (2.88%) samples were excluded as outliers (i.e., biologically implausible), resulting in a total of 4,567 blood concentrations for sulfadoxine and 4,155 for pyrimethamine available for analysis. There were 152 (18.9%) and 125 (15.6%) patients with detectable but low predose concentrations of sulfadoxine and pyrimethamine, respectively.

**TABLE 1:** Population characteristics of pharmacokinetic studies included in the present study, stratified by study and site^*a*^

Parameter	Value for indicated site in study of:									Total
	Barnes et al. (3)			Bell et al. (5)	Tekete et al. (8)	Allen et al. (9)				
	Bela Vista	Mpumalanga	Namaacha	Chileka	Bancoumana	Boane	Cutuane	Magude	Namaacha	
*n*	65	122	91	102	114	78	33	124	72	801
Sampling times	Before dosing and at days 1, 2, 3, 7, 14, 21, 28, and 42 postdosing	Before dosing and at days 1, 2, 3, 7, 14, 21, 28, and 42 postdosing	Before dosing and at days 1, 2, 3, 7, 14, 21, 28, and 42 postdosing	Before dosing and at days 2, 3, 7, 14, and 28 postdosing	Before dosing and at days 1, 3, 7, 14, 21, and 28 postdosing	Before dosing and at days 1, 2, 3, 7, 14, 21, 28, and 42 postdosing	Before dosing and at days 1, 2, 3, 7, 14, 21, 28, and 42 postdosing	Before dosing and at days 1, 2, 3, 7, 14, 21, 28, and 42 postdosing	Before dosing and at days 1, 2, 3, 7, 14, 21, 28, and 42 postdosing	
Dose (no. of tablets [500 mg-25 mg SP]) for wt band (kg)										
<10	1	1	1	1/2	1/2	NA	NA	NA	NA	
10–14	1	1	1	3/4	3/4	1	1	1	1	
15	1	1	1	1	3/4	1	1	1	1	
16–20	1	1	1	1	1	1	1	1	1	
21–22	2	2	2	1 1/4	1 1/4	2	2	2	2	
23–35	2	2	2	NA	NA	2	2	2	2	
36–40	2	2	2	NA	NA	3	3	3	3	
>40	3	3	3	NA	NA	3	3	3	3	
Sex (no. [%] of males)	30 (46)	72 (59)	48 (53)	57 (56)	63 (55)	31 (40)	18 (54)	45 (36)	34 (47)	398 (50)
Age (yr) (no. [%] of individuals)										
<2	0 (0)	0 (0)	19 (20)	0 (0)	0 (0)	0 (0)	0 (0)	0 (0)	13 (18)	32 (4)
>2–5	38 (58)	2 (1.6)	25 (22)	102 (100)	114 (100)	14 (18)	11 (33)	48 (39)	29 (35)	383 (47)
>5–20	17 (26)	66 (54)	20 (19)	0 (0)	0 (0)	23 (29)	17 (52)	44 (35)	10 (13)	197 (25)
20+	10 (15)	54 (44)	27 (30)	0 (0)	0 (0)	41 (52)	5 (15)	32 (26)	20 (28)	189 (24)
No. (%) of individuals in treatment arm										
SP	65 (100)	122 (100)	91 (100)	28 (27)	41 (36)	28 (36)	17 (52)	63 (51)	35 (49)	490 (61)
SP + AQ	0 (0)	0 (0)	0 (0)	20 (20)	40 (35)	0 (0)	0 (0)	0 (0)	0 (0)	60 (7)
SP + CQ	0 (0)	0 (0)	0 (0)	26 (25)	0 (0)	0 (0)	0 (0)	0 (0)	0 (0)	26 (3)
SP + AR	0 (0)	0 (0)	0 (0)	28 (27)	33 (29)	50 (64)	16 (48)	61 (49)	37 (51)	225 (28)
No. (%) of children under 5 years of age with nutrition score										
Normal	36 (95)	2 (100)	43 (98)	86 (85)	102 (89)	7 (50)	11 (100)	29 (60)	29 (100)	326 (85)
−3 ≤ Z-score < −2	2 (5)	0 (0)	0 (0)	15 (14)	7 (6)	4 (28)	0 (0)	13 (27)	0 (0)	41 (11)
Z-score < −3	0 (0)	0 (0)	1 (2)	1 (1)	5 (5)	3 (22)	0 (0)	6 (13)	0 (0)	16 (4)
Median (IQR) wt (kg)	15 (12–37)	50 (32–58)	26 (13–55)	11 (9–12)	14 (11–16)	55 (32–63)	32 (15–45)	25 (14–50)	15 (12–54)	18 (12–50)
Median (IQR) baseline hemoglobin (g/dl)	11 (10–12)	12 (11–13)	11 (10–12)	9 (8–10)	11 (9–12)	11 (10–14)	12 (11–13)	11 (10–12)	11 (9–13)	11 (10–12)
Geometric mean (95% range) baseline parasitemia (counts/μl)	16,028 (2,140–96,995)	22,522 (1,870–169,992)	19,874 (2,065–211,999)	52,717 (2,364–212,647)	41,694 (7,282–138,275)	5,719 (62–99,646)	768 (41–64,200)	1,853 (15–143,277)	12,561 (82–290,493)	21,700 (4,532–63,083)

a SP, sulfadoxine-pyrimethamine; AQ, amodiaquine; CQ, chloroquine; AR, artesunate; IQR, interquartile range; NA, not available.

### Pharmacokinetic model.

**FIG 1:**
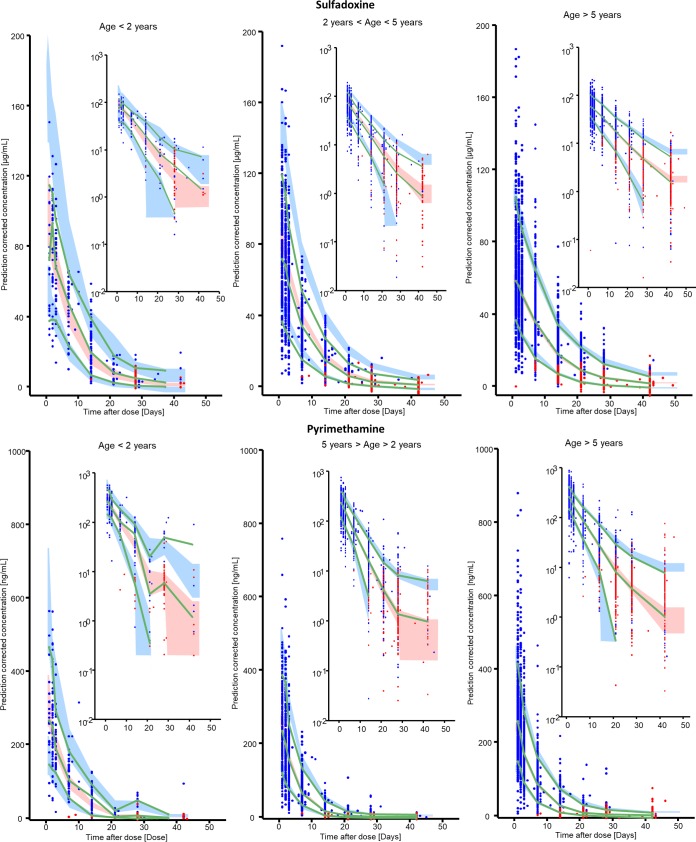
Prediction-corrected visual predictive checks for the combined final model, stratified by drug and age category. The prediction-corrected concentrations are plotted as blue dots, while the green lines represent the 5th, 50th, and 95th percentiles of the prediction-corrected concentrations. The red dots denote censored values (values below 5 μg/ml for sulfadoxine and 50 ng/ml for pyrimethamine for Chileka and values below 10 μg/ml for sulfadoxine and 10 ng/ml for pyrimethamine for all other sites) in the data set; values in the plot were simulated by the model. The shaded areas represent the 90% confidence intervals for the same percentiles, as predicted by the model.

A one-compartment model with first-order absorption and elimination provided the best fit for both sulfadoxine and pyrimethamine. The combined model for both sulfadoxine and pyrimethamine supported between-subject variability (BSV) in clearance (CL), volume of distribution, absorption rate constant, and bioavailability and a combined error structure. The final model parameter values are shown in Table 2. Prediction-corrected visual predictive checks of the observed drug concentrations versus time, to adjust for the site effects, are shown in Fig. 1. Additionally, visual predictive checks (without prediction correction) stratified by study site and age and other goodness-of-fit plots stratified by age, weight, and nutrition score are included in the supplemental material. These plots show that the median of the observed data generally fits well within the confidence interval for the 50th percentile of the model prediction for each age category, although some sites and age groups displayed more variability than others and the model sometimes over- or underpredicted the extreme percentiles. The model simulations used for dose optimization were therefore performed with the parameter values for the reference site—which contained the most patients (55% for sulfadoxine and 53% for pyrimethamine)—and by targeting of median values, which were more consistently well predicted.

**TABLE 2:** Parameter estimates for final combined sulfadoxine-pyrimethamine population pharmacokinetic model^*f*^

Parameter	Sulfadoxine		Pyrimethamine	
	Estimate	RSE (%)^*a*^	Estimate	RSE (%)^*a*^
*F*	1 fixed		1 fixed	
CL/*F* (liters/h)^*b*^	0.0264	3	0.829	3
*V*/*F* (liters)^*b*^	5.29	2	91.4	3
*k_a_* (/h)	0.521	16	1.40	80
Change in *F* for each point in Z-score below −2 (%)	−15.3	31	−26.7	13
PGA_50_ (mo after conception)	8.12	56	11.9	13
γ (Hill coefficient)	3.20	21	3.01	46
Difference from clearance in reference 5 (%)			−54.9	4
Scaling on observations at site(s) (%)				
Bancoumana, Bela Vista, Catuane^*e*^			20.2	19
Namaacha^*e*^			−22.0	23
Mpumalanga, Boane, Namaacha^*d*^	−39.7	5		
BSV (%)^*c*^ in:				
*F*	38.4	12	36.1	4
*k_a_*	126	21	171	25
*V*	11.2	23	15.5	14
CL	33.9	5	29.0	5
Correlation in CL of the two drugs (%)	60.0	6	60.0	6
Additive error (μg/ml for sulfadoxine; ng/ml for pyrimethamine)	3.79	5	6.58	5
Proportional error (%)	17.1	3	23.2	2

a Calculated from the Fisher information determined by stochastic approximation.

b Clearance and volume were allometrically scaled with total body weight, centered on the median body weight (18 kg).

c BSV values were assumed to be log-normally distributed and are reported here as approximate percent coefficients of variation (CV%).

d The reference group for scaling on observations for sulfadoxine included Magude, Bancoumana, Bela Vista, Catuane, and Chileka.

e The reference group for scaling on observations for pyrimethamine included Magude, Mpumalanga, Boane, and Chileka.

f RSE, relative standard error; *F*, relative bioavailability; CL/*F*, elimination clearance for a fully matured child; *V*/*F*, apparent volume of distribution; *k_a_*, first-order absorption rate constant; PGA_50_, the PGA at which CL is 50% of the mature value; BSV, between-subject variability.

Allometric scaling with total body weight improved the model fit substantially (change in −2× log likelihood [Δ−2LL] = 320 for sulfadoxine and 855 for pyrimethamine) and decreased BSV in the volume of distribution for both drugs. Maturation of clearance improved the model fit and decreased the −2LL by 47 points (for sulfadoxine, Δ−2LL = 15, df = 2, and *P* < 0.001; for pyrimethamine, Δ−2LL = 32, df = 2, and *P* < 0.001). Malnutrition, characterized by a low weight-for-age Z-score, was found to affect bioavailability (for sulfadoxine, Δ−2LL = 21, df = 2, and *P* < 0.001; for pyrimethamine, Δ−2LL = 81, df = 2, and *P* < 0.001), with children who had Z-scores of −3 having 15.3% and 26.7% lower bioavailabilities of sulfadoxine and pyrimethamine, respectively, than children with Z-scores of ≥−2.

Even after adjusting for body size, maturation, and nutrition score, significant site-specific differences between the pharmacokinetic profiles remained. For sulfadoxine, group A (Mpumalanga, Boane, and Namaacha) had 39.7% lower observed concentrations than those for the reference group (Magude, Bancoumana, Bela Vista, Catuane, and Chileka) (Δ−2LL = 263; df = 1; *P* < 0.001). For pyrimethamine, group B (Catuane, Bancoumana, and Bela Vista) had 22% higher observed concentrations (Δ−2LL = 1,342; df = 1; *P* < 0.001) and group C (Namaacha) 20.2% lower observed concentrations (Δ−2LL = 183; df = 1; *P* < 0.001) than those for the reference group (Magude, Mpumalanga, Boane, and Chileka). The study of Bell et al. (5) showed a 54.9% lower pyrimethamine clearance (Δ−2LL = 3,668; df = 1; *P* < 0.001) than those for the other sites. No other predefined covariates (sex, baseline hemoglobin, dose [milligrams per kilogram of body weight], concomitant medications, and baseline parasitemia) were found to be significant, and these were therefore excluded from the model.

The simulated (*n* = 500) median day 7 concentrations (*C*_day7_) for a typical 50-kg patient were 81.7 μg/ml for sulfadoxine and 132 ng/ml for pyrimethamine after standard WHO-recommended dosing. Efficacy targets were fixed to 75% of these values, i.e., 61.3 μg/ml for sulfadoxine and 98.9 ng/ml for pyrimethamine. The simulations also revealed that median maximum concentrations (*C*_max_) for a typical 10-kg patient (highest *C*_max_ among the weight bands with good representation in our clinical data) were 263 μg/ml for sulfadoxine and 785 ng/ml for pyrimethamine, with toxicity thresholds of 329 μg/ml and 981 ng/ml, respectively (125% of the median values).

**FIG 2:**
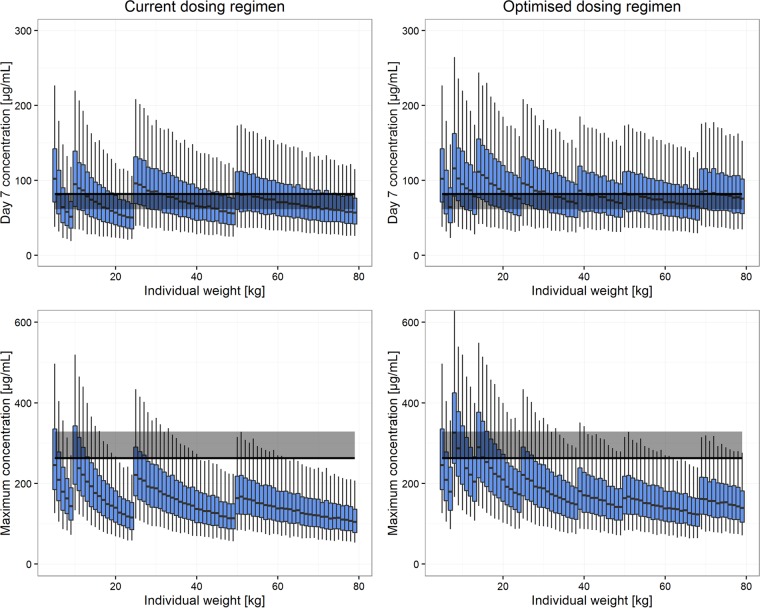
Sulfadoxine exposure. Current WHO dosing recommendations (left) are compared to optimized dosing recommendations (right). In the top panels, total exposure is represented by the drug concentration at day 7 (*C*_day7_) for patients with different body weights. The solid black line and gray band represent the median and 75% of the median of *C*_day7_, respectively, for the adult dosed with the highest dose (milligrams per kilogram). Maximum concentrations (*C*_max_) for patients with different body weights are shown in the bottom panels. The solid black line and gray band represent the highest median *C*_max_ and 125% of the highest median *C*_max_, respectively, among the well-observed population (7 to 79 kg).

**FIG 3:**
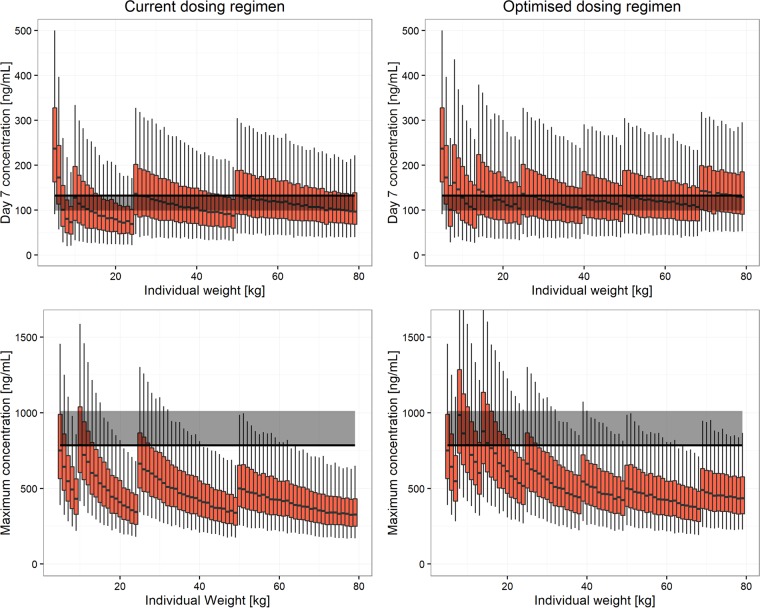
Pyrimethamine exposure. Current WHO dosing recommendations (left) are compared to optimized dosing recommendations (right). In the top panels, total exposure is represented by the drug concentration at day 7 (*C*_day7_) for patients with different body weights. The solid black line and gray band represent the median and 75% of the median of *C*_day7_, respectively, for the adult dosed with the highest dose (milligrams per kilogram). Maximum concentrations (*C*_max_) for patients with different body weights are shown in the bottom panels. The solid black line and gray band represent the highest median *C*_max_ and 125% of the highest median *C*_max_, respectively, among the well-observed population (7 to 79 kg).

Under the current dosing recommendations, simulated median sulfadoxine *C*_day7_ values for patients weighing 8 to 9, 19 to 24, 46 to 49, and 74 to 79 kg were lower than the efficacy target for sulfadoxine, while for pyrimethamine this occurred for the weight bands of 8 to 9, 14 to 24, and 42 to 49 kg. Optimized weight-based dosing using a maximum of five weight bands, with patients given 0.5, 1, 1.5, 2, 3, and 4 tablets of 500 mg sulfadoxine-25 mg pyrimethamine, was simulated. The optimized doses that achieved a median *C*_day7_ higher than the efficacy target and a median *C*_max_ lower than the toxicity threshold are shown alongside the current WHO dosing regimen in Table 3 and in Fig. 2 and 3.

**TABLE 3:** Dose optimization simulations

No. of tablets (500 mg/25 mg SP)	SP dose (mg)	wt band (kg)	
		Current WHO dosing recommendation^*a*^	Optimized dosing recommendation (for patients older than 1 yr)
0.5	250/12.5	5–9	<8
1	500/25	10–24	8–13
1.5	750/37.5		14–24
2	1,000/50	25–49	25–38
2.5	1,250/62.5	≥	39–49
3	1,500/75	≥50	50–68
4	2,000/100		≥69

a From reference 30.

**FIG 4:**
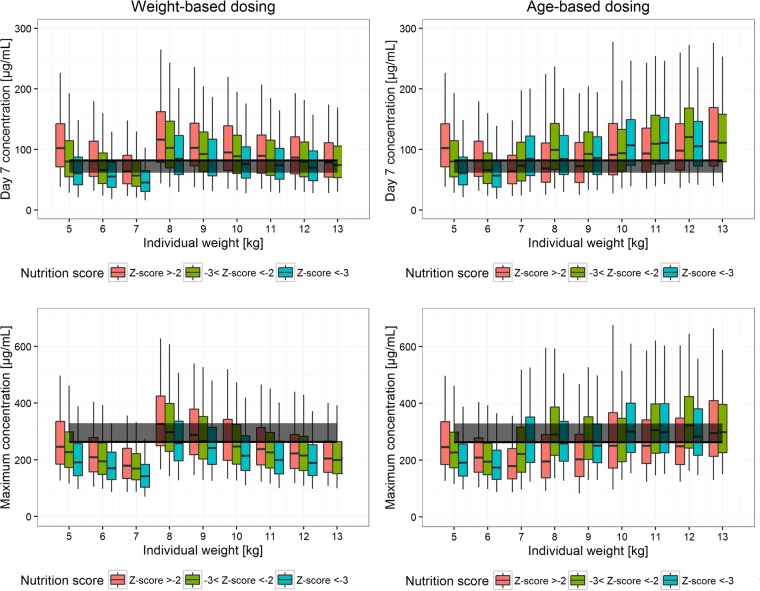
Sulfadoxine exposure. Weight-based optimized dosing recommendations (left) are compared to age-based optimized dosing recommendations (right) for young children (those with weights of <13 kg), stratified by nutrition score. In the top panels, total exposure is represented by the drug concentration at day 7 (*C*_day7_) for patients with different body weights. The solid black line and gray band represent the median and 75% of the median of *C*_day7_, respectively, for the adult dosed with the highest dose (milligrams per kilogram). Maximum concentrations (*C*_max_) for patients with different body weights are shown in the bottom panels. The solid black line and gray band represent the highest median *C*_max_ and 125% of the highest median *C*_max_, respectively, among the well-observed population (7 to 79 kg).

**FIG 5:**
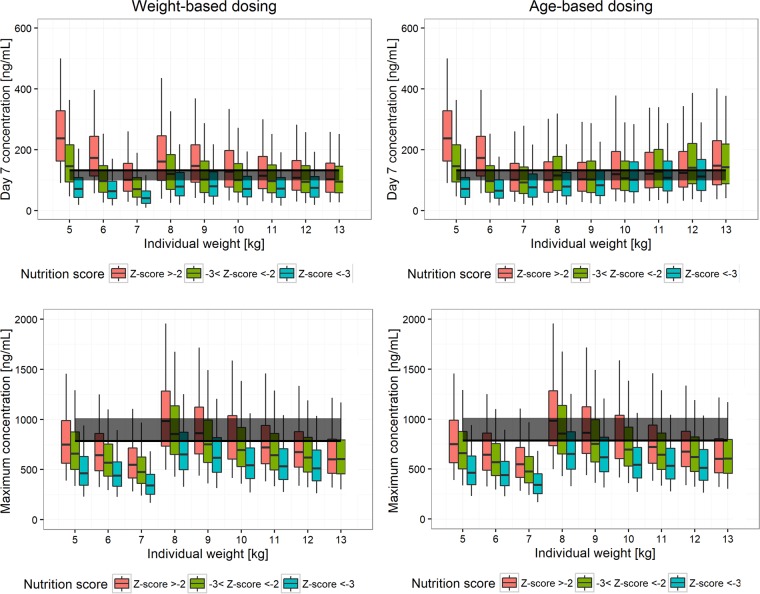
Pyrimethamine exposure. Weight-based optimized dosing recommendations (left) are compared to age-based optimized dosing recommendations (right) for young children (those with weights of <13 kg), stratified by nutrition score. In the top panels, total exposure is represented by the drug concentration at day 7 (*C*_day7_) for patients with different body weights. The solid black line and gray band represent the median and 75% of the median of *C*_day7_, respectively, for the adult dosed with the highest dose (milligrams per kilogram). Maximum concentrations (*C*_max_) for patients with different body weights are shown in the bottom panels. The solid black line and gray band represent the highest median *C*_max_ and 125% of the highest median *C*_max_, respectively, among the well-observed population (7 to 79 kg).

Under current dosing recommendations, among children with a body weight of 10 kg, moderate malnutrition (Z-score between −2 and −3) resulted in 6.68% and 21.9% lower median *C*_day7_ values for sulfadoxine and pyrimethamine, respectively, while severe malnutrition (Z-score of <−3) resulted in 20.3% and 44.3% lower median *C*_day7_ values for sulfadoxine and pyrimethamine, respectively (see Fig. S2 in the supplemental material). Thus, an alternative age-based dosing regimen for children under the age of 5 years was explored. A comparison of the weight-based and age-based dosing regimens, optimized to achieve a median *C*_day7_ higher than the efficacy target and a median *C*_max_ lower than the toxicity threshold for each weight or age band, is shown in Table 4, with simulated *C*_day7_ and *C*_max_ values for the optimized weight-based and age-based dosing regimens presented in Fig. 4 and 5.

**TABLE 4:** Optimized dosing proposals for patients under 60 months of age

Dosing proposal basis	wt (kg)	Age (mo)	No. of tablets (500 mg/25 mg SP)	SP dose (mg)
wt	<8		0.5	250/12.5
	8–13		1	500/25
	14–25		1.5	750/37.5
Age		<16	0.5	250/12.5
		16–41	1	500/25
		42–60	1.5	750/37.5

## DISCUSSION

In this study, a population pharmacokinetic nonlinear mixed-effects model was used to analyze data pooled from four studies at eight study sites. The overall aims were to describe the pharmacokinetic properties of sulfadoxine and pyrimethamine in pediatric and adult malaria patients, characterize the effects of clinical and demographic covariates, and design an optimized dosing regimen. The most significant differences between adults and children were found to be body size (accounted for by use of allometric scaling) and age, which affects the maturation of organ function. Additionally, children who were underweight for their age had lower bioavailability than that of adequately nourished children. Simulation-based predictions revealed that patients with body weights of 8 to 9, 19 to 24, 46 to 49, and 74 to 79 kg for sulfadoxine and 8 to 9, 14 to 24, and 42 to 49 kg for pyrimethamine did not reach the chosen efficacy target with the current dosing recommendation. Based on the model, a revised dosing regimen was devised and is expected to provide therapeutic exposures in small children similar to those in adults, which may improve malaria treatment in children under the age of 5 years and provide insight into dosing for chemoprevention in children.

To our knowledge, this study is the largest analysis of the pharmacokinetics of sulfadoxine-pyrimethamine to date (3, 5, 8–12), pooling data from four different clinical studies, for a total of 8,722 pharmacokinetic samples from 801 patients in Africa, among whom 415 were children. Pooling of these data empowers the study to address novel research questions and detect new covariate effects (13) for which the single studies were not adequately powered, such as the effect of malnutrition. Our results can be used to inform treatment regimens in children, one of the most vulnerable groups affected by malaria (1).

The final model has estimated parameters with values comparable to those in previous studies (Table 5), with high precision and acceptable model diagnostics. Allometric scaling significantly explained some of the differences between adults and children by accounting for changes in body size. No information on patient height was available, so we could not attempt to adjust for body composition and test fat-free mass for scaling, which may arguably be a better predictor of the size of drug-metabolizing organs (14). The absence of height information for children was more of a limitation in trying to determine the nutritional status of the children. We could only calculate weight-for-age Z-scores as a measure of nutritional status, and no attempt could be made to distinguish between stunting and wasting. Children who were underweight for their age were found to have lower bioavailability than that of adequately nourished children in the study. This may be due to decreased or delayed absorption of the drugs, or possibly to increased total body water, lower albumin levels, or other pathophysiological changes observed in malnourished children (15). As both sulfadoxine and pyrimethamine are highly protein bound (90%), lower levels of albumin may cause higher free drug concentrations, resulting in higher clearance and a larger volume of distribution, which would have the same effect on the PK profile as a decrease in bioavailability. However, changes in protein binding are not expected to affect unbound drug levels, so no dose adjustment would be necessary.

**TABLE 5:** Other published studies of sulfadoxine-pyrimethamine pharmacokinetic data for patients with uncomplicated malaria^*a*^

Parameter or characteristic	Value or description for the study of:					
	Sarikabhuti et al. (39) (median [range])	Hellgren et al. (40) (median [range])	Winstanley et al. (41) (mean)	Bustos et al. (42) (median [range])	Dzinjalamala et al. (11) (mean)	Obua et al. (10) (mean [range])
Assay	Bratton-Marshall	HPLC	HPLC	HPLC	HPLC	HPLC
Patient group	Adults with malaria	Children (8–14 yr) with malaria	Children with malaria	Adults with malaria	Children (1–12 yr) with malaria	Children (2–5 yr) with malaria
No. of participants	Responders: 5	10	8	Sulfadoxine group: 19	ACPR group: 49	55
	Nonresponders: 7			Pyrimethamine group: 13	LTF group: 66	
Sample type	Plasma	Capillary whole blood	Plasma	Serum	Capillary whole-blood spots on filter paper	Capillary whole-blood spots on filter paper
Sulfadoxine statistics						
Dose	500 mg	29.4 (25.0–35.7) mg/kg	25 mg/kg	1,500 mg	ACPR group: 34.7 mg/kg	500 mg
					LTF group: 32.3 mg/kg	
*C*_max_ (μg/ml)	Responders: 160 (151–176)	94 (78–103)	79	169 (124–279)	ACPR group: 79	171 (85–249)
	Nonresponders: 192 (143–243)				LTF group: 69	
AUC (μg/ml/h)	Responders: 45,792 (34,656–61,560)	23,064	20,016	66,192 (42,480–93,552)	ACPR group: 22,368	16,900 (2,840–27,500)^*b*^
	Nonresponders: 43,392 (32,256–55,344)	(13,176–28,992)			LTF group: 21,312	
*t*_1/2_ (h)	Responders: 228 (165.6–273.6)	214 (125–242)	115.2	261.6 (158.4–321.6)	ACPR group: 172	98 (18–177)
	Nonresponders: 184 (172.8–252)				LTF group: 154	
Pyrimethamine statistics						
Dose			1.25 mg/kg	75 mg		
*C*_max_ (ng/ml)			533	591 (173–815)		
AUC (ng/ml/h)			62,568	72,696 (28,584–161,904)		
*t*_1/2_ (h)			81.6	69.6 (38.4–302.4)		

a ACPR, adequate clinical and parasitological response; LTF, late treatment failure; *C*_max_, maximum concentration; AUC, area under the concentration-time curve; *t*_1/2_, elimination half-life; HPLC, high-pressure liquid chromatography.

b AUC from 0 to 336 h.

The inclusion of allometric scaling could not fully explain the pharmacokinetics in children younger than 2 years of age due to the significant maturation of drug-clearing organs during this time (16, 17). This was described in the model by the inclusion of a maturation function accounting for the fact that young children have a lower clearance than that of adults, after adjusting for the effect of body size. The model estimated the age (months after conception [postgestational age]) at which maturation reaches half of its maximal value (PGA_50_) to be around 8.1 months for sulfadoxine and 11.9 months for pyrimethamine, similar to previous values reported by Salman et al. (18), i.e., 9.03 months for sulfadoxine and 10.6 months for pyrimethamine. As renal function and many hepatic enzymes are expected to reach maturity by age 2 (19), a limitation is that our study does not contain data on children under the age of 1 year. More information is therefore needed to fully assess the effect of maturation on clearance in infants.

Even after adjusting for body size, maturation, and nutrition score, significant site-specific pharmacokinetic differences remained. We could include 55% of patients for sulfadoxine and 53% of patients for pyrimethamine for the reference site, but for patients at other sites an adjustment factor was needed. Additionally, the study by Bell et al. (5) found a 54.9% lower clearance than that for the other sites. That study was the only one that assayed whole-blood liquid samples (capillary blood dried-spot samples were assayed in all the other studies), and its samples were assayed in a different lab. Thus, it was not possible to determine whether this difference can be explained by differences in matrix, assay method, and/or population-specific factors.

Young children in areas of high malaria transmission are particularly vulnerable, as immunity is acquired with age and after repeated infections (2). Malaria-induced inflammation can also cause iron-deficiency anemia in children with asymptomatic malaria (20). It is therefore important that children receive adequate doses of sulfadoxine-pyrimethamine as treatment. Appropriate drug dosing in children is particularly challenging (3), and a number of studies have reported suboptimal exposures for children receiving antimalarial treatment (3, 8, 9). The final model developed in this study demonstrated sufficient predictive performance, making it suitable for dose optimization simulations. It was therefore used to simulate sulfadoxine-pyrimethamine exposures for different body weights with the current dosing guidelines and to develop optimized weight-based and age-based dosing regimens. A proposed optimized dosing regimen based on simulations is provided for the range of weights of 5 to 79 kg. The weight bands were designed using the currently available tablet size and allowing only multiples of half tablets. No widely accepted PK targets for either efficacy or toxicity are available for sulfadoxine or pyrimethamine, so efficacy was prioritized, since adverse reactions are infrequent and severe cutaneous toxicity is rare and idiosyncratic (and not dose related) (21). Furthermore, pyrimethamine has also been used safely in children, at doses as high as 2 mg/kg, for the treatment of toxoplasmosis (22). However, more-precise definitions of efficacy and safety thresholds would further improve, and potentially simplify, dosage recommendations.

Alternate optimized dosing by age bands rather than weight bands (Table 4) for children under 5 years of age was proposed to address the concerning finding of significantly lower sulfadoxine and pyrimethamine exposures in underweight-for-age young children, and it resulted in more satisfactory exposures. Age-based dosing shows a lot of promise for malnourished children, but further data are needed to allow definitive conclusions on optimal dosing in this doubly vulnerable population.

Although very young patients (1 to 12 months) were included in the weight-for-age data set, we did not have any data for patients below the age of 12 months in the pharmacokinetic data set used to build the model. Simulated optimized dosing for children younger than 12 months of age (125 mg-6.25 mg sulfadoxine-pyrimethamine for weights of <5 kg) was made possible by inclusion of a maturation function in the model. However, further pharmacokinetic data are needed for investigation into the maturation of clearance of sulfadoxine and pyrimethamine in children under 12 months of age to inform optimal dosing in this age group. This is reflected by the relative standard error (56% for sulfadoxine and 13% for pyrimethamine) in the parameter for postgestational age at which CL is 50% of the mature value (PGA_50_).

### Conclusions.

This study reports the largest pharmacokinetic analysis of sulfadoxine-pyrimethamine, to date, and proposes a model accounting for the effects of body size, maturation, and nutritional status. The analysis revealed suboptimal sulfadoxine-pyrimethamine exposures in some weight bands for children given the current WHO-recommended dosing regimens. Children who were underweight for their age had decreased bioavailability, with a greater effect for pyrimethamine. Accounting for all these effects, the model was used to propose an optimized sulfadoxine-pyrimethamine dosing regimen, which is essential to ensure that children, particularly malnourished children, achieve exposures similar to those in adults and thus have an equivalent likelihood of treatment success. Improved treatment success would reduce the selective pressure for the development of resistance and prolong the useful therapeutic life span of sulfadoxine-pyrimethamine.

## MATERIALS AND METHODS

All relevant published pharmacology studies were identified by searching PubMed, Embase, Google Scholar, ClinicalTrials.gov, and conference proceedings by using the key words “sulfadoxine or pyrimethamine pharmacokinetics” or “sulfadoxine or pyrimethamine concentrations” and “clinical study.” The first and last authors of identified studies were contacted and invited to join this pooled analysis by contributing individual patient data to the Worldwide Antimalarial Resistance Network (WWARN) repository as part of a study group if their studies were prospective sulfadoxine and pyrimethamine studies of nonpregnant African patients with uncomplicated P. falciparum infection, especially children under the age of 5 years. The WWARN automated data management, curation, and analysis tools converted the submitted data into a set of defined data variables in a standard format, following the WWARN clinical and pharmacology data management and statistical analysis plans (23, 24). Study reports were generated from the formatted data sets and sent back to investigators for validation or clarification. All participating authors agreed to the WWARN terms of submission (25), which ensure that all data uploaded were anonymized and obtained with informed consent and in accordance with any laws and ethical approvals applicable in the country of origin.

The pharmacokinetic data used for modeling were pooled from 4 different previously published clinical studies (3, 5, 8, 9) from the African countries of Mozambique, South Africa, Mali, and Malawi and were collected at eight different study sites. The data from the studies in Mali and Malawi were only for children, while the data from the studies in Mozambique and South Africa included both children and adults. Adults received a single dose of 1,500 mg sulfadoxine-75 mg pyrimethamine and children a minimum of 25 mg/kg sulfadoxine-1.25 mg/kg pyrimethamine, according to the weight bands shown in Table 1. Sulfadoxine-pyrimethamine was administered alone (250 children, 304 adults) or in combination with chloroquine (34 children), artesunate (85 children, 113 adults), or amodiaquine (29 children). All sulfadoxine-pyrimethamine concentrations were measured by use of capillary whole-blood dried spots on filter paper (*n* = 4,214), except in the study conducted by Bell et al., in which concentrations were measured in liquid samples of either capillary (*n* = 285) or venous (*n* = 84) whole blood. Six to nine samples per patient were collected at all sites, at least predose and on days 1, 3, 7, 14, 21, and 28.

Nonlinear mixed-effects modeling was implemented in the software Monolix Suite 2016R1 (Lixoft, France) to analyze the pharmacokinetic data, and parameters were estimated using the stochastic approximation expectation maximization (SAEM) algorithm. The pharmacokinetics of sulfadoxine and pyrimethamine were first modeled independently to determine their structural model and covariate effects, and the models were then combined into one model to investigate possible correlations between the pharmacokinetic parameters of the two drugs. One-, two-, and three-compartment disposition models with first-order absorption were evaluated for the structural model. Between-subject variability (BSV) of the pharmacokinetic parameters was evaluated by assuming a log-normal distribution. A combined error model with both additive and proportional components was used for the residual unexplained variability (RUV). The −2× log-likelihood (−2LL) values, goodness-of-fit plots, visual predictive checks (*n* = 1,000), residual error plots, and the Wald test guided the model development.

All concentration results were available as the original values reported by the analytical laboratory assay, including the readings below the lower limit of quantification (LLOQ), except for the data collected at 2 sites in Mozambique, for which all concentrations lower than 10 ng/ml for pyrimethamine and 10 μg/ml for sulfadoxine were censored and reported as below the LLOQ (BLQ). These censored values were handled by use of the M3 approach suggested by Beal (26), using the censoring functionality in Monolix. All other BLQ readings were used in the model as the original values reported by the laboratory to make the best use of the data. Drug concentration samples were collected before dosing to determine if any drug from the previous treatment was still present in the circulation. If detectable values were found in these predose samples, it was assumed that the pharmacokinetic profile was in the terminal elimination phase, and all the disposition compartments in the model were initialized to the observed drug concentration. Biologically implausible samples were identified and excluded using a model-based approach in which values with extreme normalized prediction distribution errors (NPDEs) for both drugs were discarded.

The effects of weight, age, nutritional status (measured as the weight-for-age Z-score; unfortunately, no data on height or mid-upper-arm circumference were available), study site, sex, baseline hemoglobin, total dose (milligrams per kilogram), concomitant medications, baseline parasitemia, and sample blood matrix were tested as predefined covariates.

The effect of body size was taken into account by using allometric scaling (16) with total body weight to adjust all volumes with an exponent of 1 and flow rates (clearance and flow rates to and from peripheral compartments) with an exponent of 0.75 (27). Unfortunately, no height information was available for the patients, so testing of alternative size descriptors, such as fat-free mass, or adjustments for BMI were not possible. The effect of age on clearance (16) was tested using a sigmoidal maturation (MAT) function of postgestational age, as follows: MAT = PGA^γ^/(PGA^γ^ + PGA_50_^γ^), where PGA is the postgestational age, PGA_50_ is the PGA at which clearance is 50% of the mature value, and γ is the Hill coefficient determining the steepness of the curve.

The nutritional status of children was determined based on weight-for-age Z-scores calculated using the R macro and igrowup.standard function provided on the WHO website (28). The weight-for-age Z-scores were determined from growth curves developed by the WHO Multicenter Growth Reference Study (MGRS). That study was undertaken between 1997 and 2003 to generate new growth curves for assessing the growth and development of infants and young children around the world. The MGRS collected primary growth data and related information from approximately 8,500 children from widely different ethnic backgrounds and cultural settings (Brazil, Ghana, India, Norway, Oman, and the United States). Children were considered malnourished if they had a Z-score of <−2 (29). This effect was added to the model by using a “hockey stick” model according to the following formula: effect = (change in bioavailability per unit change in Z-score) × (Z-score + 2).

For other categorical covariates (study site, sex, concomitant medications, and sample blood matrix), one reference subcategory (REF) was defined, and relative differences from REF were calculated for each of the other subcategories. For other continuous covariates (baseline hemoglobin, dose [milligrams per kilogram], and baseline parasitemia), the linear covariate effects on the log-transformed PK parameters were explored, centered on the median value in the population in order to incorporate the central tendency of the data, as follows: log *P_i_* = log *P*_pop_ + β × log(*cov*/*m*) + η_*i*_, where *P_i_* is the individual parameter value, *P*_pop_ is the population parameter value, β is the covariate effect, *cov* is the continuous covariate, *m* is the median value, and η_*i*_ is the random effect for parameter *P_i_*. This ensures that positive PK parameters are preserved and that the effect can be interpreted approximately as a relative change in parameter value for a unit change in the covariate. In cases of several categorical and/or continuous covariate effects on the same PK parameter, all effects were included by using a multiplicative relationship.

We screened for covariate effects by using the “full approach,” where all effects with potential impacts on sulfadoxine-pyrimethamine PK were estimated simultaneously and tested for statistical significance by use of the Wald test (*P* < 0.05). The covariate effects detected as significant with the Wald test were then included in the model by a stepwise approach. First, they were added one by one and retained if they produced a decrease in the −2LL of >3.84 for 1 df (*P* < 0.05). They were then confirmed with a backward elimination step in which each covariate-PK parameter relationship was removed one by one and retained only if an increase of >10.83 in −2LL for 1 df (*P* < 0.001) was observed.

No widely accepted PK targets for either efficacy or toxicity are available for sulfadoxine-pyrimethamine, and effective concentrations increase with the accumulation of dihydrofolate reductase and dihydropteroate synthase mutations. The clinical data set contained data on a large number of patients, among whom adults achieved high efficacy, and very little toxicity was reported for both adults and children. We therefore decided pragmatically to target the concentrations that the model predicted for the patients included in our analysis. Median values of *C*_day7_ and *C*_max_ were chosen as reference values with the same tolerance threshold. The use of the 25% tolerance margin was dictated by pragmatic considerations to accommodate for the feasibility of the suggested optimized regimen in a programmatic setting (i.e., to avoid the creation of too many weight bands and/or the breaking of tablets).

Monte Carlo simulations based on the final PK model were used to evaluate dosing regimens. The median *C*_day7_ values for a typical 50-kg patient after standard recommended dosing were simulated, and efficacy targets were fixed to 75% of these values. The median *C*_max_ values were simulated, and toxicity thresholds were fixed to 125% of the highest value among the weight bands with good representation in our clinical data. Dosing regimens were evaluated based on whether they achieved median *C*_day7_ values higher than the efficacy targets and median *C*_max_ values lower than the toxicity thresholds for patients with different body weights. We first evaluated the currently recommended WHO dosing regimen (30) and then explored alternative dosing regimens. Historical malaria patient data from several studies (31–38) and unpublished data from routine clinical monitoring of children with malaria (under 5 years old) and the patients used in this study were used to create a model describing weight-for-age values in malaria patients (see the supplemental material). This model was used to simulate plausible weight-for-age values, as follows. Twenty *in silico* patients were generated for each kilogram of weight from 5 to 80 kg. For weight bands that contained children under the age of 5 years, for which age-for-weight Z-scores are defined, 40 more patients per kg (20 with Z-scores of <−3 and 20 where −3 ≤ Z-score < −2) were simulated (60 patients in total per body weight [in kilograms]). None of the simulated patients had Z-scores of <−4.27, as no patients in the studies pooled for our PK analysis had a Z-score of <−4.27. The resulting database had 1,880 *in silico* malaria patients with ages between 1 and 50 years. The current WHO dosing guidelines were simulated in 500 hypothetical clinical trials, using the 1,880 *in silico* patients and the final developed population PK model.

## Supplementary Material

Supplemental material
