# Supplementary material for: Population Pharmacokinetic Properties of Sulfadoxine and Pyrimethamine: a Pooled Analysis To Inform Optimal Dosing in African Children with Uncomplicated Malaria
Source: Antimicrob Agents Chemother. 2018 Apr 26;62(5):e01370-17. doi: 10.1128/AAC.01370-17 (PMC5923181; doi:10.1128/AAC.01370-17)
Supplement: Supplemental material [file supp_62_5_e01370-17__index.html]

Supplemental material 

# Population Pharmacokinetic Properties of Sulfadoxine and Pyrimethamine: a Pooled Analysis To Inform Optimal Dosing in African Children with Uncomplicated Malaria

## Supplemental material

- Supplemental file 1 -

  Supplemental material

  PDF, 1.3M
