# Supplementary material for: Population Pharmacokinetic Properties of Sulfadoxine and Pyrimethamine: a Pooled Analysis To Inform Optimal Dosing in African Children with Uncomplicated Malaria
Source: Antimicrob Agents Chemother. 2018 Apr 26;62(5):e01370-17. doi: 10.1128/AAC.01370-17 (PMC5923181; doi:10.1128/AAC.01370-17)
Supplement: Supplemental material [file AAC.01370-17_zac005187091s1.pdf]

Supplementary material

Historical malaria patient data from several studies (1-8), and unpublished data from routine clinical monitoring of malaria infected children (under five years) patients and the patients used in this study, were used to create a model describing weight for age in malaria patients. This model was used to simulate plausible body weight for malaria patients in the *in silico* database. A piecewise linear model fitted the data well with a combined error structure. The parameter values of the model are given in Table S1 and the visual predictive check is shown in Figure S1.

Table S1: Parameter values of Age for Weight model.

**Parameter**	**Estimate**	**%RSE[a]**
Weight at 6 months [kg]	5.64	12
Slope 1	3.84	29
Break point 1 [years]	0.573	14
Slope 2	2.22	11
Break point 2 [years]	1.99	21
Slope 3	1.7	5
Additive error	1.1	16
Proportional error (%)	10.5	12

RSE, relative standard error; Slope 1, slope of line for age <= 1.99 years; Break point 1, Estimated age at which line breaks; Slope 2, slope of line for 1.99< age <= 5 years.

RSE (%) is calculated from the Fisher information determined by stochastic approximation.

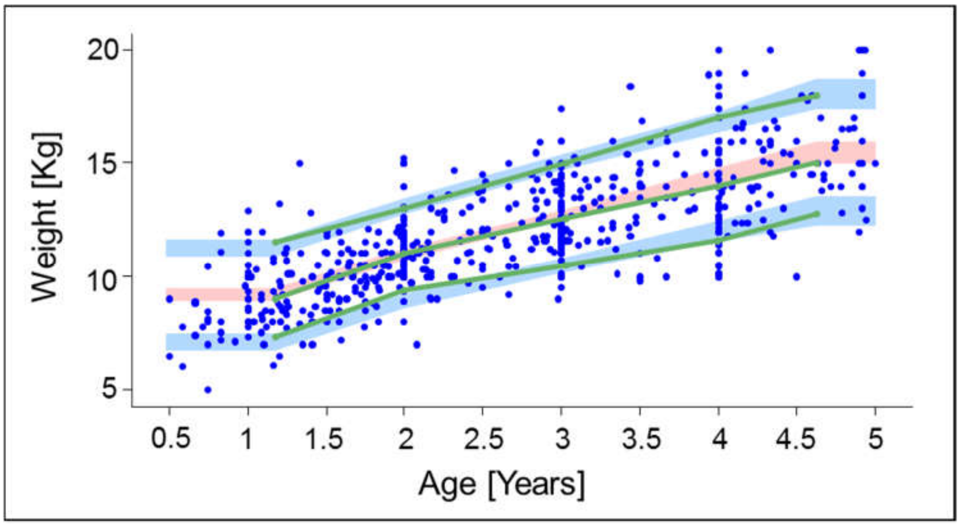

Figure S1: Visual predictive check for the Age for Weight model. The empirical percentile is plotted as green lines, representing the 5th, 50th, and 95th percentiles of the observed data. The shaded areas represent the 95% confidence intervals for the same percentiles, as predicted by the model.

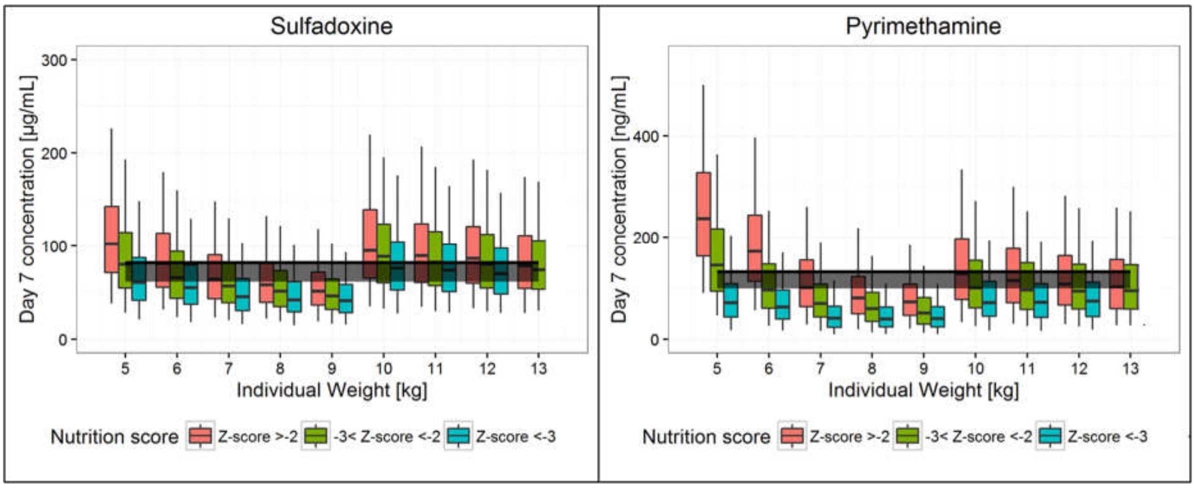

Figure S2: Exposure showing the effect of malnutrition: Current WHO dosing recommendations for sulfadoxine (left) and pyrimethamine (right), stratified by z-score. Total exposure is represented by drug concentration at day 7 (C_{day7}) for patients (< 5 years) with different body-weights. The solid black line and grey band represent the median and 75% of the median of C_{day7}, respectively, for the adult dosed with the highest mg/kg.

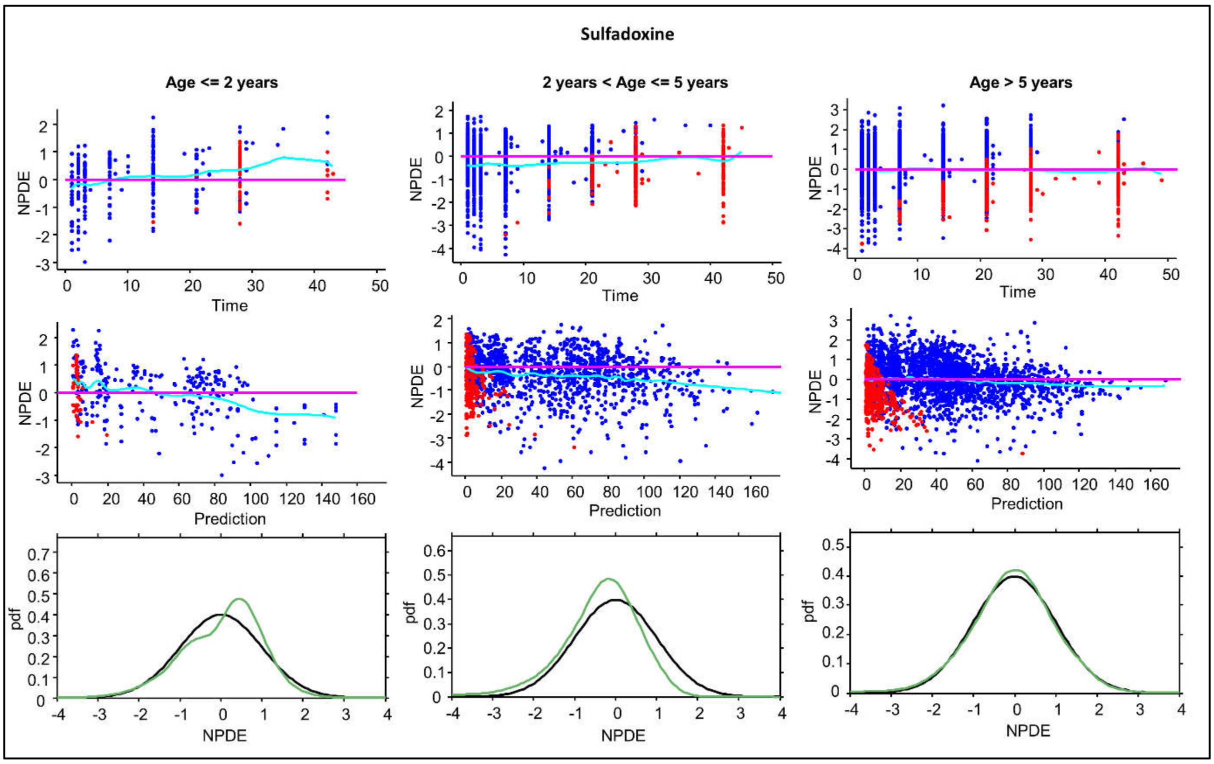

Figure S3: NPDE plots for sulfadoxine stratified by age. Blue dots represent observed data and red dots represent censored data simulated by the model.

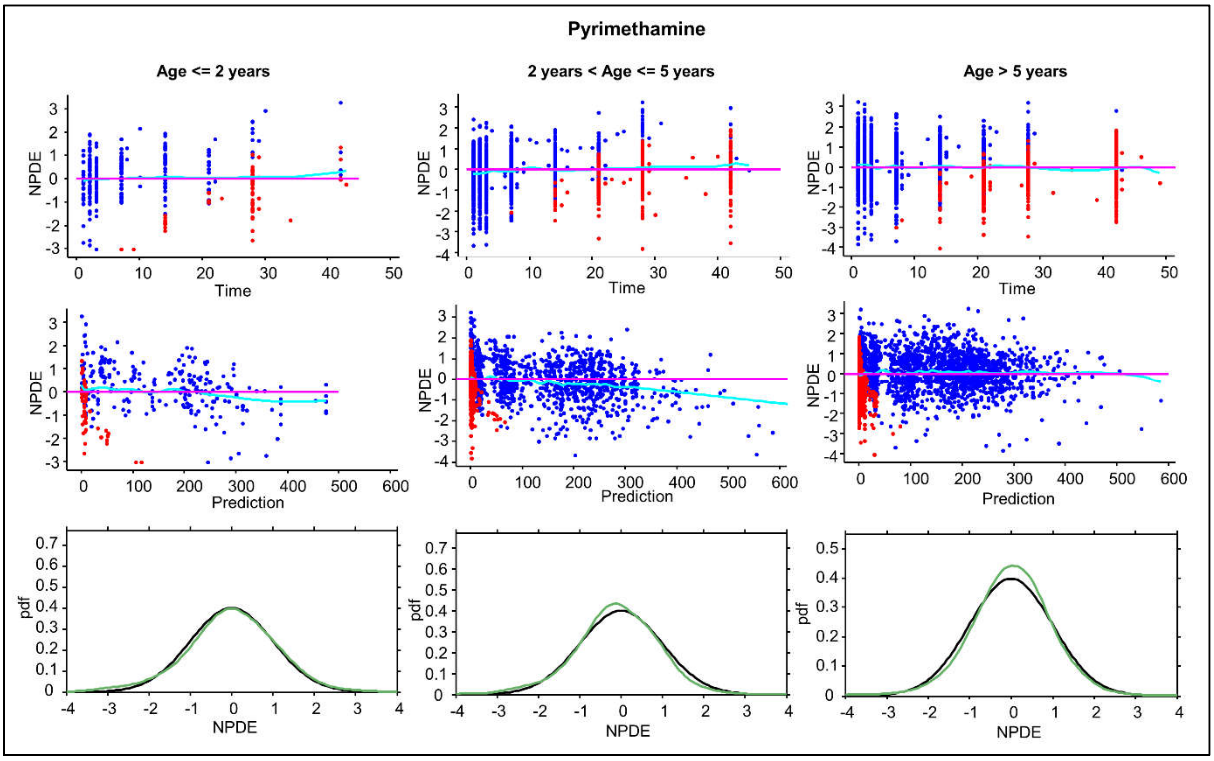

Figure S4: NPDE plots for pyrimethamine stratified by age. Blue dots represent observed data and red dots represent censored data simulated by the model.

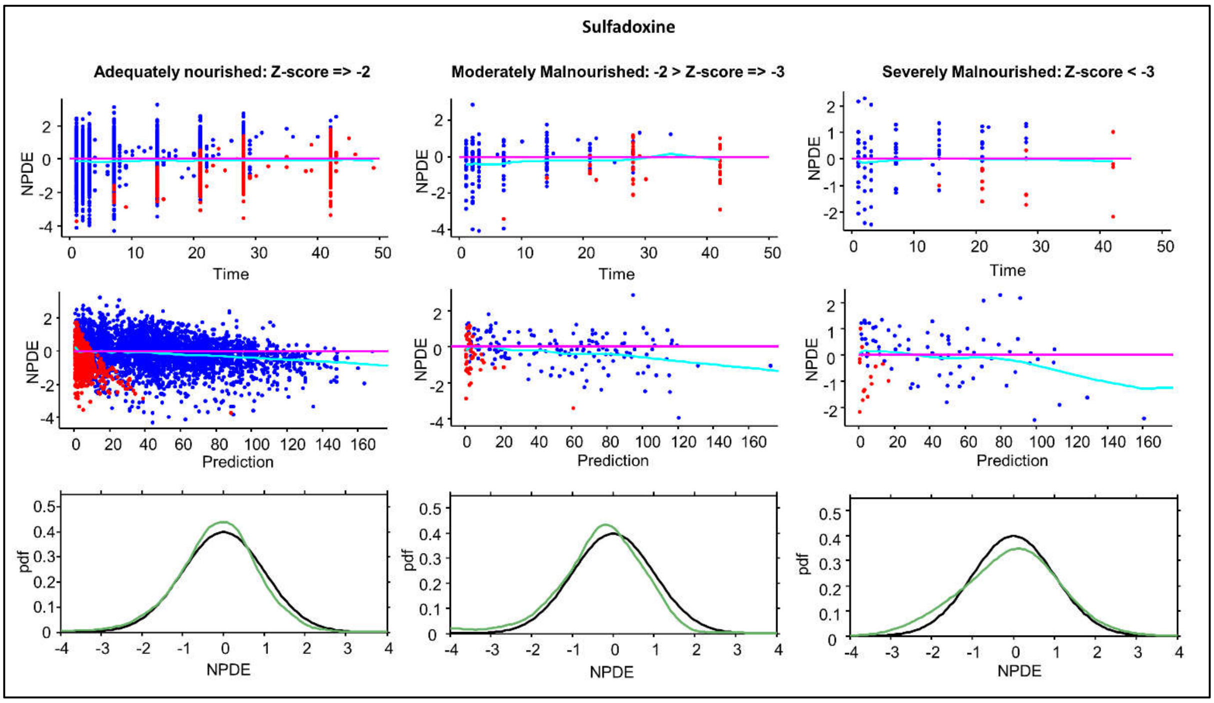

Figure S5: NPDE plots for sulfadoxine stratified by nutrition score. Blue dots represent observed data and red dots represent censored data simulated by the model.

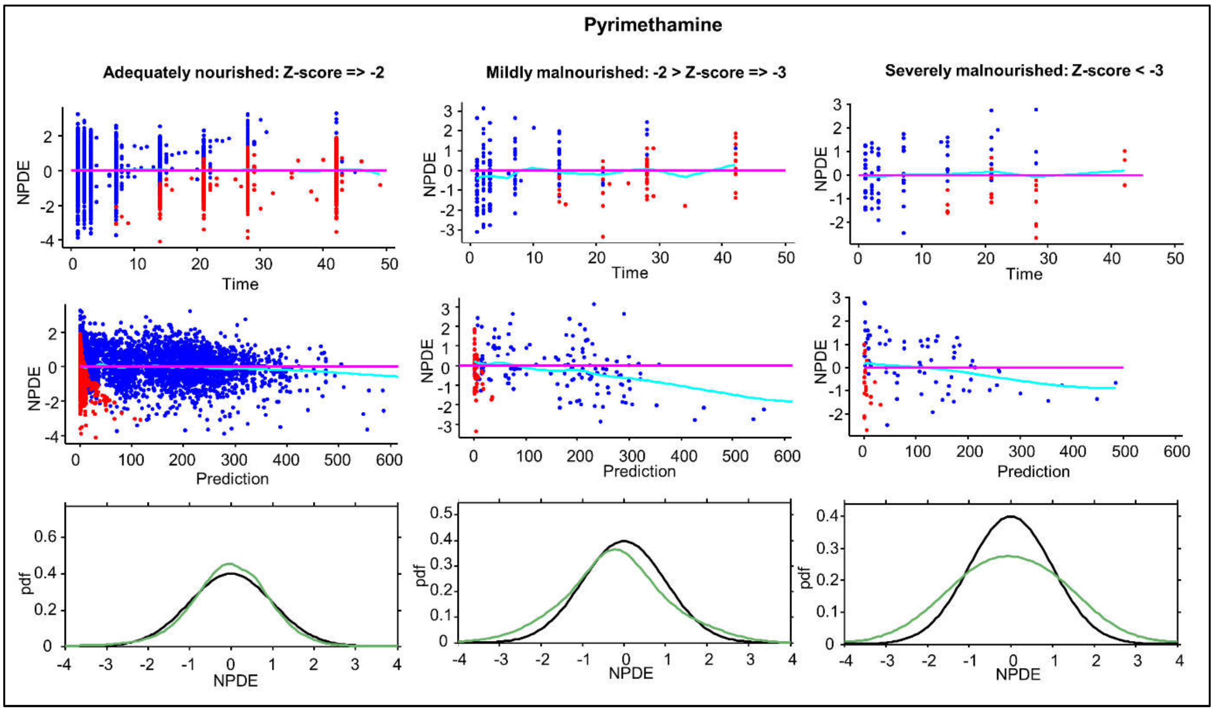

Figure S6: NPDE plots for pyrimethamine stratified by nutrition score. Blue dots represent observed data and red dots represent censored data simulated by the model.

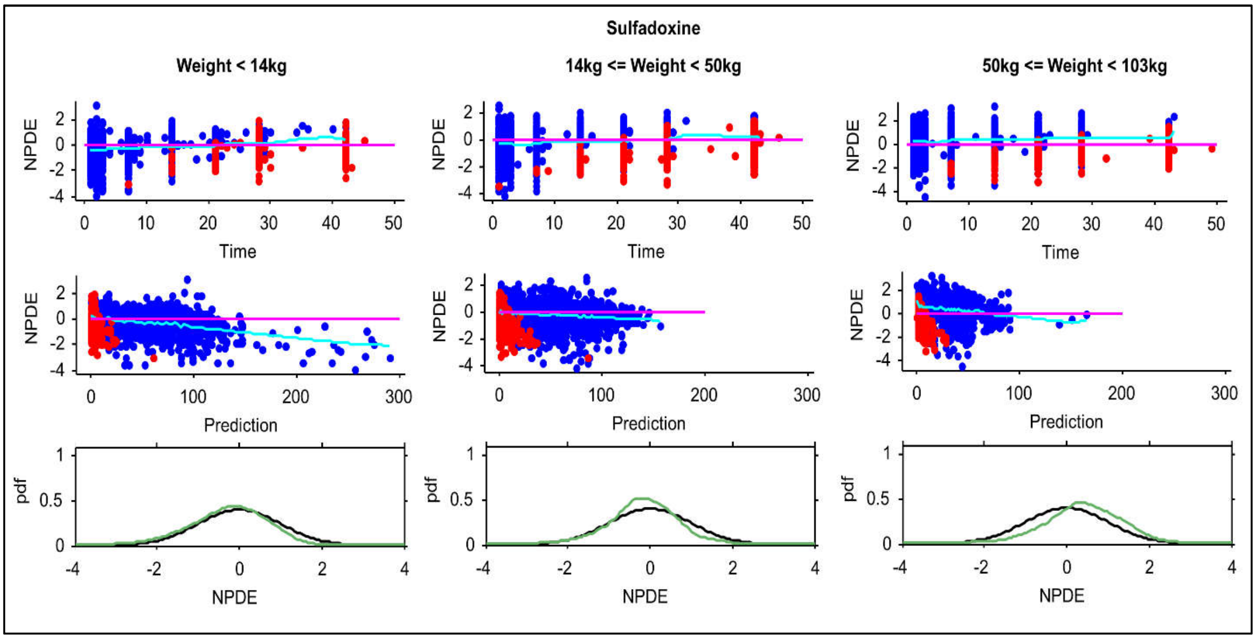

Figure S7: NPDE plots for sulfadoxine stratified by weight. Blue dots represent observed data and red dots represent censored data simulated by the model.

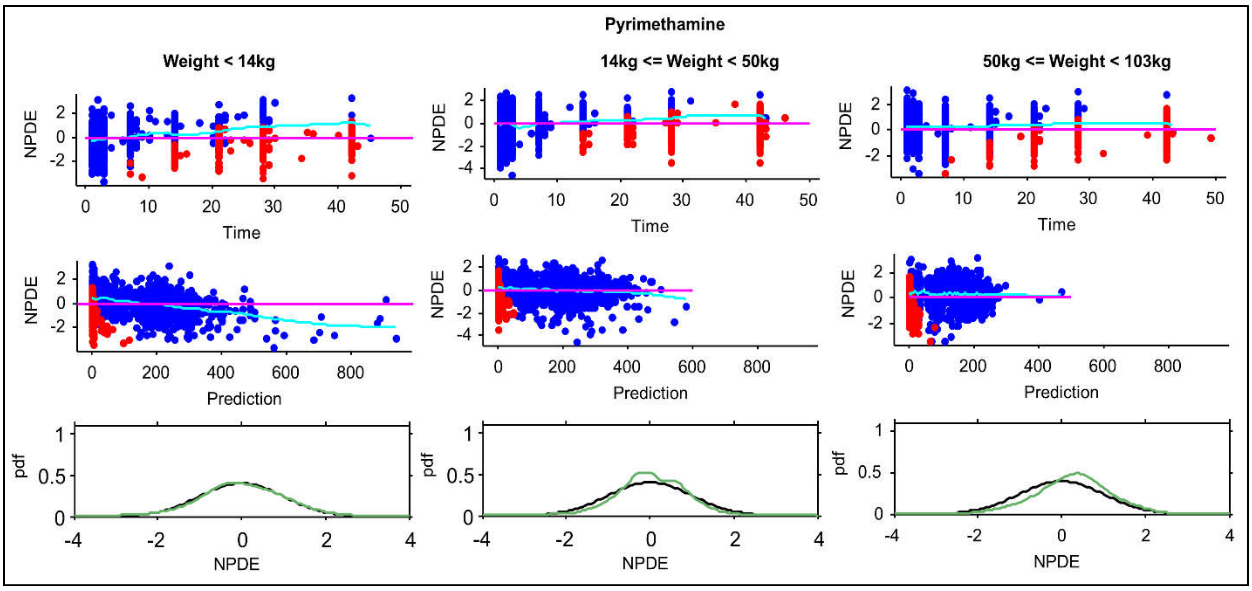

Figure S8: NPDE plots for pyrimethamine stratified by weight. Blue dots represent observed data and red dots represent censored data simulated by the model.

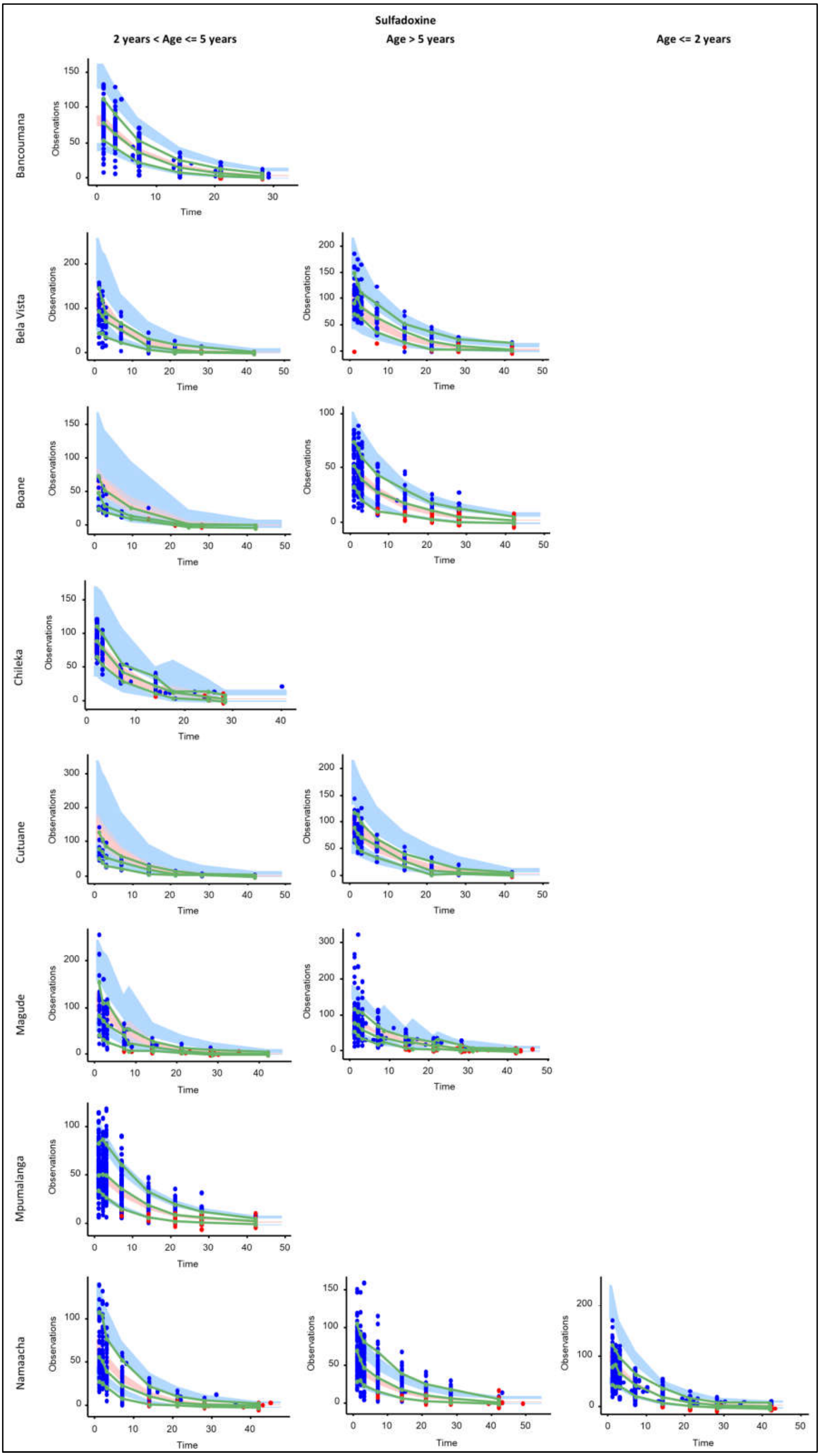
Sulfadoxine
2 years < Age <= 5 years
Age > 5 years
Age <= 2 years
Bancoumana
Bela Vista
Boane
Chileka
Cutuane
Magude
Mpumalanga
Namaacha
Observations
Time

Figure S9: Figure 1: Visual Predictive Checks for sulfadoxine, stratified by site, and age category. The observations are plotted as blue dots while the green lines represent the 5th, 50th, and 95th percentiles of the observed data. The red dots denote censored values (values below, Chileka: 5 µg/mL for sulfadoxine and 50 ng/mL for pyrimethamine, all other sites: 10 µg/mL for sulfadoxine and 10 ng/mL for pyrimethamine) in the dataset, values in the plot are simulated by the model. The shaded areas represent the 90% confidence intervals for the same percentiles, as predicted by the model.

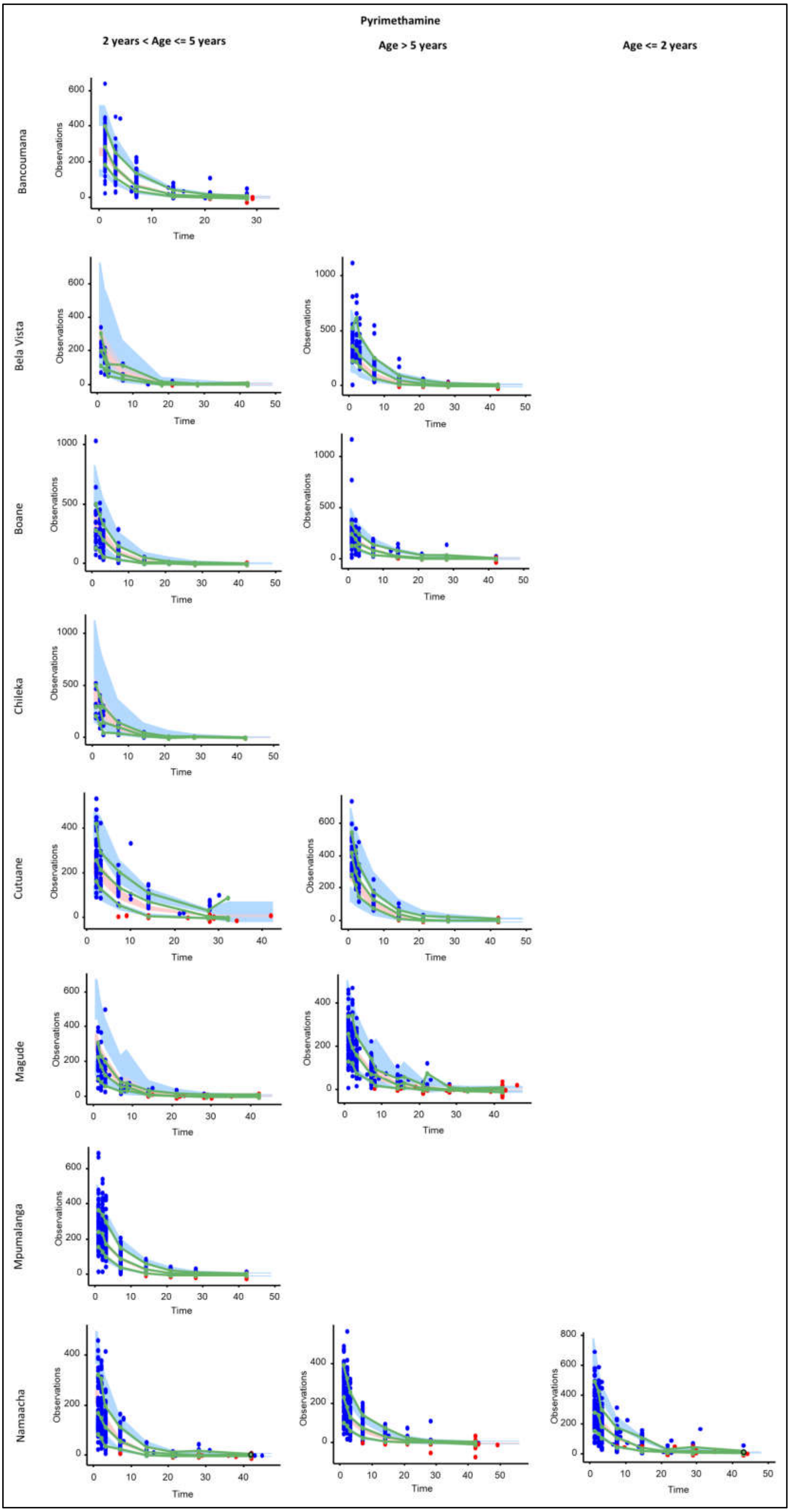

Pyrimethamine
2 years < Age <= 5 years
Age > 5 years
Age <= 2 years
Bancoumana
Bela Vista
Boane
Chileka
Cutuane
Magude
Mpumalanga
Namaacha
Observations
Time

Figure S10: Figure 1: Visual Predictive Checks for pyrimethamine, stratified by site, and age category. The observations are plotted as blue dots while the green lines represent the 5th, 50th, and 95th percentiles of the observed data. The red dots denote censored values (values below, Chileka: 5 µg/mL for sulfadoxine and 50 ng/mL for pyrimethamine, all other sites: 10 µg/mL for sulfadoxine and 10 ng/mL for pyrimethamine) in the dataset, values in the plot are simulated by the model. The shaded areas represent the 90% confidence intervals for the same percentiles, as predicted by the model.

References

1. Tarning J, Chotsiri P, Jullien V, Rijken MJ, Bergstrand M, Cammas M, McGready R, Singhasivanon P, Day NPJ, White NJ, Nosten F, Lindegardh N. 2012. Population pharmacokinetic and pharmacodynamic modeling of amodiaquine and desethylamodiaquine in women with Plasmodium vivax malaria during and after pregnancy. Antimicrob Agents Chemother 56:5764–73.

2. Rijken MJ, McGready R, Jullien V, Tarning J, Lindegardh N, Phyo AP, Win AK, Hsi P, Cammas M, Singhasivanon P, White NJ, Nosten F. 2011. Pharmacokinetics of amodiaquine and desethylamodiaquine in pregnant and postpartum women with Plasmodium vivax malaria. Antimicrob Agents Chemother 55:4338–4342.

3. Stepniewska K, Taylor W, Sirima SB, Ouedraogo EB, Ouedraogo A, Gansané A, Simpson J a, Morgan CC, White NJ, Kiechel J-R. 2009. Population pharmacokinetics of artesunate and amodiaquine in African children. Malar J 8:200.

4. Jullien V, Ogutu B, Juma E, Carn G, Obonyo C, Kiechel J-R. 2010. Population pharmacokinetics and pharmacodynamic considerations of amodiaquine and desethylamodiaquine in Kenyan adults with uncomplicated malaria receiving artesunate-amodiaquine combination therapy. Antimicrob Agents Chemother 54:2611–7.

5. Mwesigwa J, Parikh S, McGee B, German P, Drysdale T, Kalyango JN, Clark TD, Dorsey G, Lindegardh N, Annerberg A, Rosenthal PJ, Kamya MR, Aweeka F. 2010. Pharmacokinetics of

artemether-lumefantrine and artesunate-amodiaquine in children in Kampala, Uganda. Antimicrob Agents Chemother 54:52–9.

6. Faucher J-F, Aubouy A, Adeothy A, Cottrell G, Doritchamou J, Gourmel B, Houzé P, Kossou H, Amedome H, Massougbodji A, Cot M, Deloron P. 2009. Comparison of sulfadoxine-pyrimethamine, unsupervised artemether-lumefantrine, and unsupervised artesunate-amodiaquine fixed-dose formulation for uncomplicated plasmodium falciparum malaria in Benin: a randomized effectiveness noninferiority trial. J Infect Dis 200:57–65.

7. Schramm B, Valeh P, Baudin E, Mazinda CS, Smith R, Pinoges L, Sundaygar T, Zolia YM, Jones JJ, Comte E, Bruneel A, Branger M, Jullien V, Carn G, Kiechel JR, Ashley EA, Guérin PJ. 2013. Tolerability and safety of artesunate-amodiaquine and artemether-lumefantrine fixed dose combinations for the treatment of uncomplicated Plasmodium falciparum malaria: two open-label, randomized trials in Nimba County, Liberia. Malar J 12:250.

8. Adjei GO, Kristensen K, Goka BQ, Hoegberg LCG, Alifrangis M, Rodrigues OP, Kurtzhals JAL. 2008. Effect of concomitant artesunate administration and cytochrome P4502C8 polymorphisms on the pharmacokinetics of amodiaquine in Ghanaian children with uncomplicated malaria. Antimicrob Agents Chemother 52:4400-4406
